# Association between vancomycin trough concentration monitoring and gestational age in neonates: a single-center, retrospective observational study

**DOI:** 10.1186/s12887-026-07630-8

**Published:** 2026-09-04

**Authors:** Hui Chen, Xing Liu, Kanglu Ju, Xiao Song, Kai Zhao, Zeyang Wei, Bo Guo

**Affiliations:** Department of Pharmacy, Northwest Women’s and Children’s Hospital, Xi’an, China

**Keywords:** Neonate, Vancomycin, Trough concentration monitoring, Gestational age, Individualized medication

## Abstract

**Background:**

The vancomycin dosing regimen for neonates is primarily determined by gestational age (GA) and postnatal age(PNA). Nonetheless, initial trough concentrations frequently fall below therapeutic levels in neonates administered vancomycin. This study seeks to examine the relationship between vancomycin trough concentrations and gestational age in neonates, thereby offering a reference for individualized vancomycin dosing tailored to neonates of varying gestational ages.

**Methods:**

A retrospective observational study was undertaken to collect clinical data on neonates treated with vancomycin and subjected to trough concentration monitoring in the Neonatal Intensive Care Unit (NICU) at Northwest Women’s and Children’s Hospital over the preceding six years.

**Results:**

Utilizing a two-piecewise linear regression model, the P-value for the log-likelihood ratio was 0.007, indicating statistical significance, and an inflection point was identified at 34.6 weeks(approximately 242 days). For gestational ages less than 34.6 weeks, a positive correlation was observed between GA and the initial vancomycin trough concentration (TCV) (β = 0.08, 95% CI: 0.01, 0.14, *p* = 0.021). Conversely, for gestational ages exceeding 34.6 weeks, a negative trend of borderline significance (β = -0.10, 95% CI: -0.20 to 0.00, *P* = 0.059).

**Conclusion:**

Vancomycin trough concentrations in neonates exhibit a significant correlation with GA. This retrospective study, conducted at a single center, revealed a non-linear relationship between gestational age and initial vancomycin trough concentrations in neonates, identifying an inflection point at approximately 34.6 weeks. The results indicate that existing gestational age-based dosing protocols may inadequately account for pharmacokinetic transitions, underscoring the necessity for prospective studies to validate therapeutic drug monitoring protocols stratified by gestational age.

## Introduction

Vancomycin, a glycopeptide antibiotic, exhibits potent antibacterial activity against Gram - positive bacteria, especially against multidrug - resistant pathogens like methicillin - resistant Staphylococcus aureus (MRSA) and coagulase - negative staphylococci (CoNS). It is one of the key agents for treating severe Gram - positive bacterial infections in the neonatal intensive care unit (NICU) [1–3].

The 2020 guidelines from the Infectious Diseases Society of America (IDSA) aim to identify the most effective pharmacokinetic/pharmacodynamic (PK/PD) parameters for vancomycin therapy. For adult patients, a 24 - hour area under the concentration - time curve to minimum inhibitory concentration ratio (AUC_24_/MIC) exceeding 400 is regarded as the best predictor of therapeutic efficacy. Neonates, especially those born preterm, exhibit underdeveloped physiological functions. Distinct differences in renal function, body composition, and plasma protein concentrations, among other developmental characteristics, set them apart from adults and older children. These differences result in unique and complex pharmacokinetic and pharmacodynamic processes for vancomycin in this population [1].

Considering that neonates may exhibit distinct and variable pharmacokinetic parameters, the guidelines advocate for therapeutic drug monitoring (TDM), identifying trough concentration as the preferred indicator for monitoring.

Vancomycin possesses a narrow therapeutic window, wherein elevated serum concentrations are associated with an increased risk of adverse effects, including nephrotoxicity and ototoxicity. Conversely, subtherapeutic concentrations may lead to treatment failure and enhance the potential for the development of bacterial resistance [1]. Consequently, it is imperative to monitor serum concentrations, particularly trough levels, in neonates receiving vancomycin treatment. Adjusting the dosing regimen based on these results to achieve individualized therapy is crucial for ensuring therapeutic efficacy and minimizing adverse reactions. In China, the empirical use of vancomycin for suspected early-onset sepsis is more prevalent than in North America, especially within tertiary neonatal intensive care units (NICUs) that experience elevated local incidences of multidrug-resistant Staphylococcus aureus and coagulase-negative staphylococci. National consensus guidelines endorse this approach for high-risk neonates exhibiting severe clinical presentations. This practice accounts for the early initiation of vancomycin (median day 1) observed in our cohort.

Research indicates that the initial trough concentration of vancomycin (TCV) is associated with variables such as postnatal age(PNA), gestational age(GA), body weight, estimated glomerular filtration rate (eGFR), and serum albumin levels [4]. Furthermore, factors predictive of vancomycin clearance, including body size, renal function, and various age metrics (postnatal, gestational, and postmenstrual age), have been validated [5–8]. Current evidence suggests that body weight, postmenstrual age, and serum creatinine levels are significant covariates influencing vancomycin clearance (CL) and volume of distribution (V) in neonatal population pharmacokinetic (PPK) models [9, 10].

Gestational age plays a pivotal role in neonatal physiological development and drug metabolism. Notable variations in renal function, volume of distribution, and clearance are observed among neonates of differing gestational ages, which can significantly influence vancomycin concentrations. At present, while numerous studies have been conducted on the monitoring of vancomycin trough concentrations in neonates, there is a relative paucity of systematic research examining the impact of varying gestational ages on these trough levels.

This study conducted a retrospective analysis of clinical data from neonates who received vancomycin treatment, with the objective of investigating the association between vancomycin trough concentrations and gestational age. The findings aim to elucidate gestational age-specific patterns in vancomycin trough concentrations, thereby establishing an evidence-based foundation for gestational age-stratified dosing regimens and therapeutic drug monitoring (TDM) protocols.

## Methods

This retrospective observational study was conducted at Northwest Women’s and Children’s Hospital in China, encompassing patients who underwent vancomycin therapy between October 2018 and July 2024. The study population comprised individuals admitted to either the neonatal general ward or the neonatal intensive care unit. Exclusion criteria included the absence of available vancomycin concentration data, a vancomycin treatment duration of less than 48 h, administration of vancomycin beyond the first 28 days of life, or termination of treatment due to mortality. This study was approved by the Medical Ethics Committee of Northwest Women’s and Children’s Hospital (No. 2023010). The rationale for employing a retrospective design in this study is grounded in the pharmacokinetic challenges associated with vancomycin administration in neonates, who exhibit a narrow therapeutic window. The attainment of initial trough concentrations is influenced by various developmental factors. Notably, gestational age serves as a composite indicator of organ maturation, particularly renal maturation and body composition. However, its independent and potentially non-linear impact on trough concentrations has not been systematically assessed, especially after adjusting for confounding variables such as body weight at the initiation of vancomycin therapy, renal function, and the vancomycin dosing regimen. The dosing regimen in this cohort includes: (i) single dose (mg/kg), (ii) dosing interval (hours), (iii) total daily dose (mg/kg/day), and (iv) route of administration, which is exclusively intravenous. The total daily dose, calculated as (single dose × 24) ÷ dosing interval, was utilized as the primary exposure variable in regression models, as it encapsulates both the magnitude and frequency of dosing. Consequently, we employed multivariable regression and non-linear modeling techniques to elucidate the relationship between gestational age and trough vancomycin concentrations, aiming to identify threshold effects that could guide dosing stratification. This study was conducted in accordance with the Declaration of Helsinki. All data were collected anonymously and analyzed retrospectively without any intervention to participants. The requirement for written informed consent was waived by the ethics committee due to the retrospective nature of this study. Clinical trial number: not applicable.

Two independent investigators extracted data from the hospital’s electronic medical records using a standardized form that had been pilot tested. They collected variables related to vancomycin pharmacokinetics in neonates, including demographic and clinical characteristics, lab biomarkers, and therapeutic indicators: gestational age, postnatal age, weight, infection type, premature rupture of membranes, vancomycin treatment duration, trough concentration, white blood cell count, neutrophil count, hemoglobin, platelet count, C-reactive protein, albumin, blood urea, and serum creatinine. Discrepancies between the investigators were resolved through discussion, with a third investigator settling any unresolved issues.

Laboratory parameters collected were measured within 24 h before vancomycin initiation, including white blood cell count (WBC), neutrophil count (NEU), hemoglobin (Hb), platelet count, C-reactive protein (CRP), albumin (ALB), urea, serum creatinine (SCr), and serum vancomycin concentrations. These laboratory tests were obtained as part of routine clinical evaluations for suspected sepsis. Specifically, CRP serves as an indicator of infection severity; platelet count and hemoglobin reflect hematological status. Urine output was not consistently documented in the electronic medical records and was thus excluded from this retrospective analysis.

The estimated glomerular filtration rate (eGFR) was calculated using the Schwartz formula: eGFR (mL/min/1.73 m²) = k × Body length (cm) ÷ SCr (mg/dL), where k = 0.45 for infants and k = 0.33 for premature infants.

Vancomycin was administered intravenously, with the dosage determined according to hospital administration protocols aligned with the neonatal subsection of the national pediatric dosing guidelines. The dosage regimen utilized in this study is detailed in Table 1.

**Table 1:** Vancomycin dosing regimen

Gestational Age (Week)	DOL (Day)	Single Dose (mg/kg)	Dose Interval (Hour)
≤ 29	0∼14	Common infection dose:10 mg/kg; Severe infection dose: 15 mg/kg	24
	> 14		12
30∼36	0∼14		12
	> 14		8
37∼44	0∼7		12
	> 7		8

*Total daily dose (mg/kg/day) = single dose × 24 ÷ dosing interval. This table presents the neonatal-specific dosing protocol derived from the neonatal subsection of the National Pediatric Dosing Guidelines

Initial trough blood samples were obtained 30 min prior to the administration of the fourth vancomycin infusion. The quantification of vancomycin blood concentrations was conducted utilizing an enzyme-multiplied immunoassay technique. (Emit ^®^ 2000; SIEMENS, Munich, Germany).

Our analytical strategy was formulated to address three sequential research questions: (1) What are the differences in patient characteristics across various trough concentration strata? This was assessed using univariate and bivariate analyses. (2) Is there an independent linear association between gestational age (GA) and initial vancomycin trough concentration (TCV) after adjusting for covariates? This was evaluated using multivariable linear regression. (3) Does this association exhibit a non-linear pattern with a threshold effect? This was examined using generalized additive models (GAM) and two-piecewise linear regression. This stepwise approach ensures that any detected non-linearity is not confounded by known determinants of vancomycin disposition. To examine variations in baseline characteristics across the spectrum of initial TCV, patients were stratified into tertiles of initial TCV (< 7.0, 7.0–10.8, > 10.8 µg/mL) solely for descriptive analysis. This data-driven strategy was employed to achieve approximately equal group sizes (*n* ≈ 47–48 per group), facilitating a robust comparison of baseline characteristics, rather than applying clinically defined thresholds that would result in highly imbalanced groups due to the observed TCV distribution in our cohort. The primary analysis considers TCV as a continuous variable; thus, the tertile stratification is not utilized for the classification of clinical efficacy.Categorical variables were evaluated using the χ² test, while normally distributed data were analyzed via one-way analysis of variance. For data with a skewed distribution, the Kruskal-Wallis H test was employed. To explore the relationship between gestational age and initial TCV, three distinct models were developed using both univariate and multivariate linear regression methodologies. These models included: an unadjusted model (without covariates), a minimally adjusted model (adjusting only for sociodemographic variables), and a fully adjusted model (incorporating all covariates listed in Table 2). The effect size and its 95% confidence interval were documented. To account for potential nonlinear relationships between gestational age and initial TCV, a generalized additive model (GAM) was utilized for curve smoothing. Additionally, a two-stage linear regression model was employed to further elucidate any nonlinearity present. To assess the robustness of the findings, a sensitivity analysis was conducted. Gestational weeks were categorized into quartiles, and a trend P-value was calculated to substantiate the results when considering gestational weeks as continuous variables, while also investigating the potential for nonlinearity. Statistical modeling was conducted using the software packages R (http://www.R-project.org, The R Foundation) and EmpowerStats (http://www.empowerstats.com, X&Y Solutions, Inc, Boston, MA). P values of less than 0.05 (two-tailed) were considered to indicate statistical significance.

**Table 2:** Baseline Characteristics of Participants

Characteristics	Total	Initial TCV, µg/mL			*P*-value
		T1 (< 7.0)	T2 (7.0-10.8)	T3(> 10.8)	
N	143	45	46	52	
Sex, N (%)					0.202
Male	88 (61.54%)	25 (55.56%)	26 (56.52%)	37 (71.15%)	
Female	55 (38.46%)	20 (44.44%)	20 (43.48%)	15 (28.85%)	
Postnatal age (DOL), Median (Q1–Q3), day	1.00 (1.00–5.50)	1.00 (1.00–2.00)	1.00 (1.00–4.00)	1.00 (1.00–6.25)	0.881
Gestational Age, Mean (SD), day	235.68 ± 35.08	229.11 ± 36.86	240.26 ± 36.03	237.31 ± 32.39	0.292
Body length, Mean (SD), cm	42.33 ± 7.36	40.23 ± 6.92	42.61 ± 7.92	43.90 ± 6.89	0.046
Weight, Median (Q1–Q3), kg	1.65 (1.16–3.25)	1.28 (0.96–2.78)	1.86 (1.24–3.46)	1.92 (1.38–3.06)	0.160
Gestational Age, N (%)					0.302
≤ 29 weeks	33 (23.08%)	14 (31.11%)	11 (23.91%)	8 (15.38%)	
>29,≤37weeks	59 (41.26%)	17 (37.78%)	16 (34.78%)	26 (50.00%)	
>37 weeks	51 (35.66%)	14 (31.11%)	19 (41.30%)	18 (34.62%)	
WBC, Median (Q1–Q3), ×10⁹/L	10.61 (7.34–15.58)	8.86 (5.84–14.02)	12.74 (8.32–17.42)	10.53 (6.90–17.26)	0.155
NEU, Median (Q1–Q3), ×10⁹/L	5.04 (2.78–9.83)	3.63 (1.95–8.58)	6.25 (3.75–9.81)	4.71 (2.79–10.36)	0.603
Hb, Mean (SD), g/L	136.94 ± 26.23	131.69 ± 20.39	141.49 ± 25.79	137.56 ± 30.46	0.204
Platelet, Median (Q1–Q3), ×10⁹/L	208.00 (86.00–319.00)	149.00 (75.00–281.00)	264.50 (160.75–332.50)	188.00 (76.75–284.75)	0.128
CRP, Median (Q1–Q3), mg/L	19.33 (5.00–47.72)	25.25 (6.51–53.51)	20.72 (5.00–38.81)	15.86 (4.20–46.63)	0.448
ALB, Mean (SD), g/L	29.61 ± 4.61	29.50 ± 4.86	30.77 ± 4.16	28.67 ± 4.63	0.077
Urea, Median (Q1–Q3), mmol/L	3.92 (2.53–5.83)	3.47 (2.41–5.74)	3.77 (2.49–6.32)	4.36 (3.30–6.10)	0.901
SCr, Mean (SD), µmol/L	37.73 ± 17.11	38.81 ± 16.27	35.05 ± 13.72	39.17 ± 20.27	0.435
eGFR, Median (Q1–Q3), mL/min/1.73 m²	37.14 (26.09–59.44)	31.89 (24.57–56.70)	46.92 (26.23–61.53)	37.70 (27.11–59.02)	0.542
Vancomycin Daily Dose, Median (Q1–Q3), mg/kg	32.76 (30.00–45.00)	27.22 (20.00–30.00)	31.74(30.00-37.50)	38.46 (30.00–45.00)	< 0.001

*Abbreviations: TCV* trough concentrations of vancomycin, *WBC* white blood cell, *NEU* Neutrophil, *Hb* Hemoglobin, *CRP* C-reactive protein, *ALB* albumin, *SCr* serum creatinine, *eGFR* estimated glomerular filtration rate

TCV tertiles (<7.0, 7.0–10.8, >10.8 μg/mL) are used for descriptive and exploratory purposes only. The primary analysis treats TCV as a continuous variable.Data availability: WBC n=143, NEU n=143, Hb n=142, platelet n=143, CRP n=143, ALB n=142, urea n=142, SCr n=143, eGFR n=143.Serum creatinine was measured within 24 hours prior to vancomycin initiation (median DOL 1, IQR 1–5.5). Values in the first days of life may partially reflect maternal renal function due to transplacental equilibration.Gestational age strata in this table (≤29 weeks, >29–≤37 weeks, >37 weeks) correspond to the dosing tiers in Table 1

## Results

### Baseline characteristics of participants

**Fig. 1:**
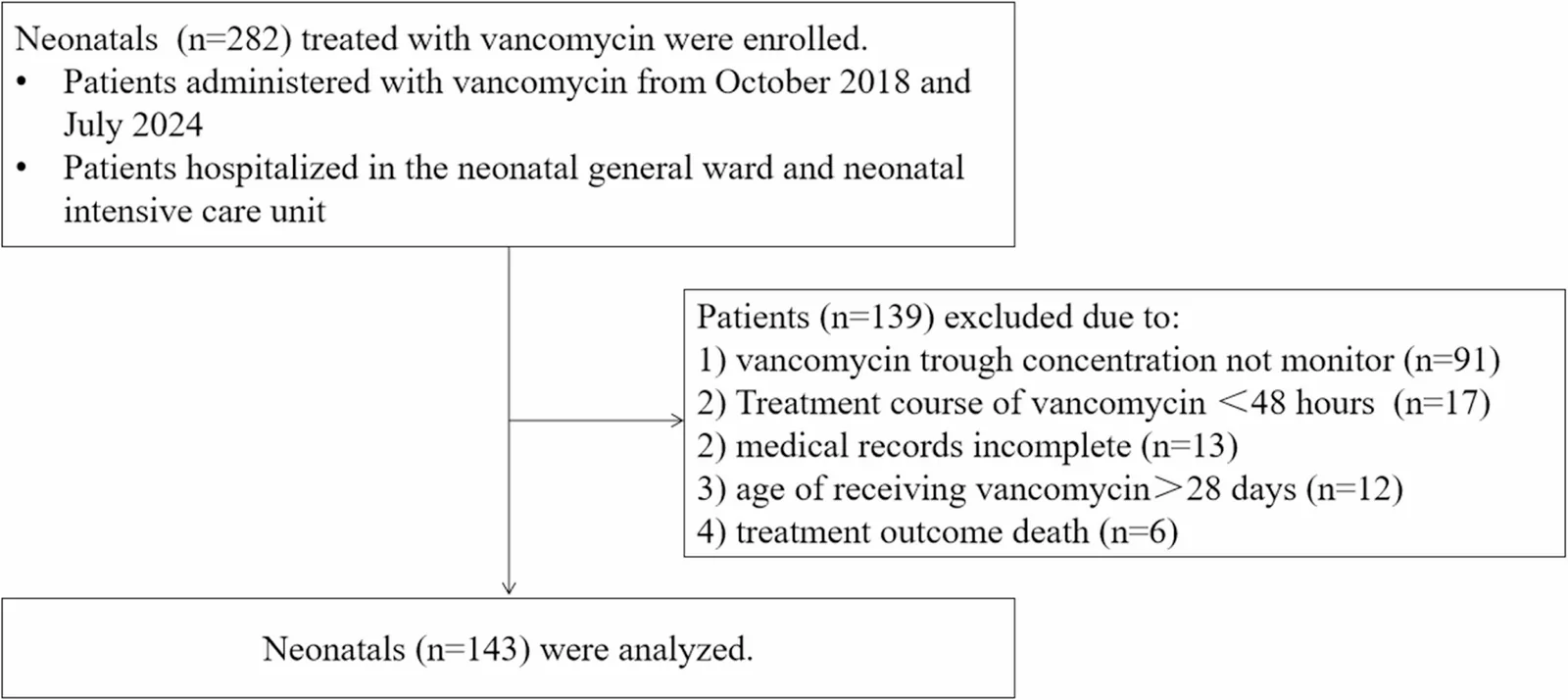
Flowchart of the study population

In this analysis, out of a total of 282 participants, 139 were deemed ineligible for the study, as depicted in Fig. 1. The exclusion criteria for these 139 individuals were as follows: 91 lacked continuous therapeutic drug monitoring of vancomycin,17 were receiving vancomycin treatment course less than 48 h,13 had incomplete medical documentation, 12 were treated with vancomycin for over 28 days, and 6 died during the treatment. All 143 patients had complete data for vancomycin concentration, GA, and dosing regimen. Laboratory parameters were available for ≥ 95% of patients; exact n per variable is shown in Table 2 footnotes. Model II used complete-case analysis (*n* = 143). The clinical profiles and laboratory findings of the 143 eligible patients are detailed in Table 2.

The median postnatal age at vancomycin initiation was 1.00 (1.00–5.50) day, and 61.54% were male. Body length and vancomycin daily dose differed significantly across initial TCV tertiles (*P* = 0.046 and *P* < 0.001, respectively); the T3 group had a higher mean body length and median daily dose compared with the T1 group. No significant differences were observed in sex, postnatal age, GA, weight, WBC, NEU, Hb, platelet count, CRP, ALB, urea, SCr, or eGFR (all *P* > 0.05).

### Univariate analysis of initial TCV

The results of the univariate analysis are presented in Table 3. The analysis revealed a positive correlation between the daily dose of vancomycin and elevated initial TCV in neonates (β = 0.23, 95% CI: 0.15 to 0.31, *P* < 0.0001). Furthermore, no significant associations were identified between initial TCV in neonates and the variables of sex, age, body length, weight, GA, WBC, CRP, ALB, SCr, and eGFR (Table 4).

**Table 3:** The Result of Univariate Analysis of Initial TCV

	Statistics	Effect Size (β)	*P*-value
Sex			
Male	88 (61.54%)	ref	
Female	55 (38.46%)	-0.47 (-2.42, 1.48)	0.636
Age	4.62 ± 6.69	0.02 (-0.12, 0.16)	0.807
Length	42.33 ± 7.36	0.09 (-0.04, 0.22)	0.175
Weight	2.15 ± 1.18	0.00 (-0.00, 0.00)	0.422
Gestational Age	235.68 ± 35.08	0.02 (-0.01, 0.05)	0.163
Vancomycin Daily Dose	32.76 ± 11.02	0.23 (0.15, 0.31)	< 0.0001
WBC	12.34 ± 7.31	0.02 (-0.11, 0.15)	0.755
CRP	37.06 ± 48.58	-0.00 (-0.02, 0.02)	0.927
ALB	29.61 ± 4.61	-0.13 (-0.33, 0.08)	0.236
SCr	37.73 ± 17.11	0.01 (-0.04, 0.07)	0.699
eGFR	47.64 ± 32.49	0.01 (-0.02, 0.04)	0.691

*Abbreviations: TCV* trough concentrations of vancomycin, *WBC* white blood cell, *CRP* C-reactive protein, *ALB* albumin, *SCr* serum creatinine, *eGFR* estimated glomerular filtration rate

**Table 4:** Relationship Between Gestational Age and Initial TCV in Different Models of Multivariate Analysis

Variable	Crude Model (β, 95% CI, *P*)	Model I (β, 95% CI, *P*)	Model II (β, 95% CI, *P*)
Gestational Age	0.02 (-0.01, 0.05) 0.163	0.04 (-0.02, 0.10) 0.169	0.02 (-0.03, 0.07) 0.405
Gestational Age quartile			
Q1	Ref	Ref	Ref
Q2	1.69 (-0.88, 4.26) 0.199	1.75 (-0.87, 4.37) 0.192	1.41 (-0.98, 3.80) 0.249
Q3	5.03 (2.41, 7.65) 0.000	6.41 (2.95, 9.87) 0.000	5.06 (1.85, 8.27) 0.003
Q4	1.72 (-0.84, 4.29) 0.190	4.12 (-0.41, 8.66) 0.078	2.94 (-1.21, 7.10) 0.167
P for trend	0.0581	0.0255	0.0782

*Abbreviations: TCV* trough concentrations of vancomycin, *ALB* albumin, *SCr* serum creatinine, *eGFR* estimated glomerular filtration rate, *CI* Confidence interval, *Ref* Reference

Crude model: No variables are adjusted. Model I: Adjusted sex, age, weight, length. Model II: Adjusted sex, age, weight, length, vancomycin daily dose, ALB, eGFR and SCr.Gestational age quartiles were defined as: Q1 (< 206 days, ~<29.4 weeks), Q2 (207–229 days, ~ 29.6–32.7 weeks), Q3 (230–273 days, ~ 32.9–39.0 weeks), Q4 (> 274 days, ~>39.1 weeks). Quartile analysis was conducted as a sensitivity analysis to test the robustness of the continuous GA–TCV relationship

### Gestational age and initial trough concentration of vancomycin: nonlinear association analysis

**Fig. 2:**
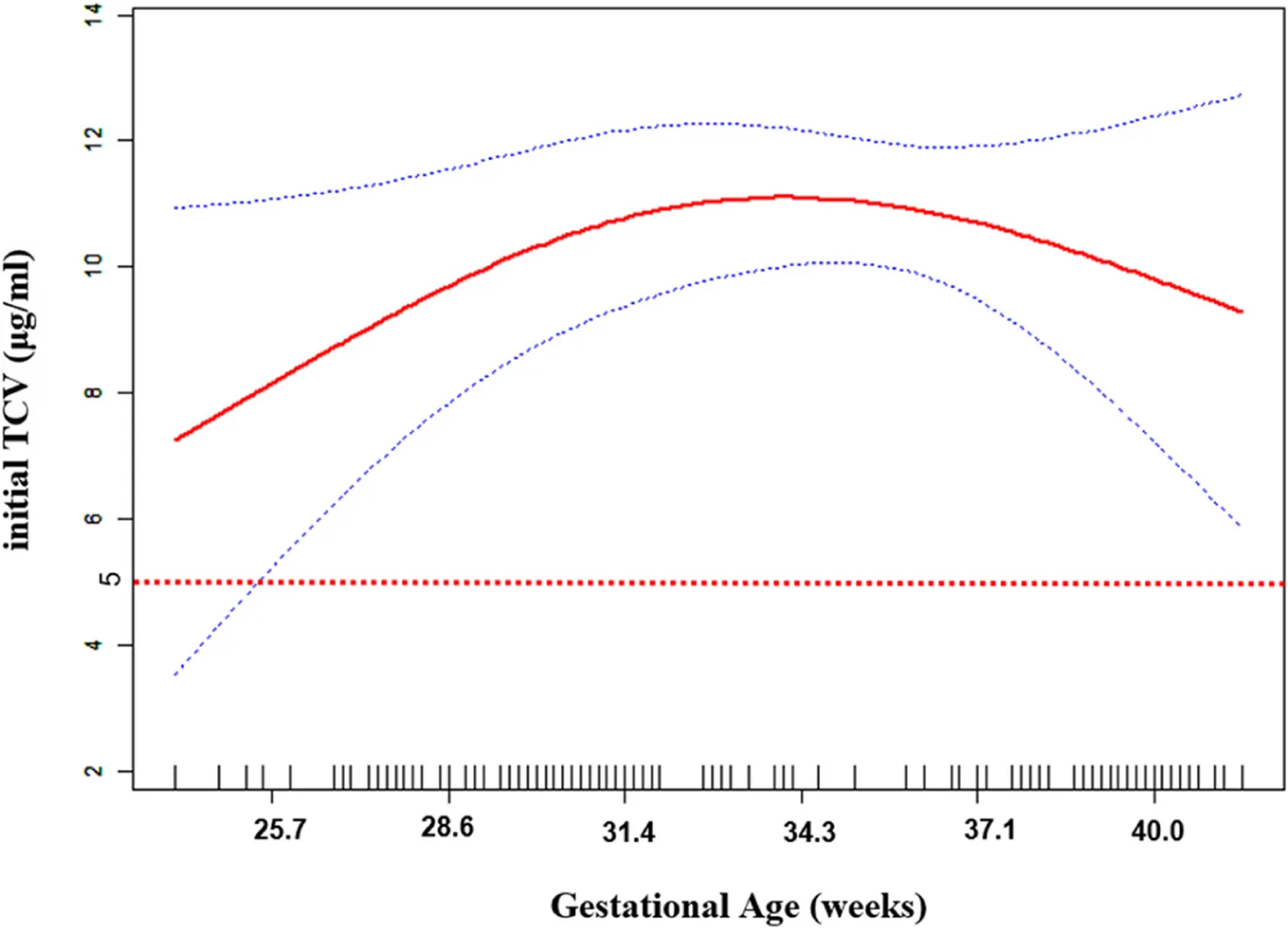
The non-linear relationship between Gestational Age and initial TCV in neonates. A non-linear association between Gestational Age and initial TCV was found in a generalized additive model (GAM). Solid red line represents the smooth curve fit between variables. Blue bands represent the 95% of confidence interval from the fit. All adjusted for sex, age, weight, length, vancomycin daily dose, ALB, eGFR and SCr

Considering gestational age (GA) was a continuous variable in this study, we adopted generalized additive model (GAM) to explore the potential nonlinear relationship between GA and initial trough concentration of vancomycin (initial TCV). After adjusting for confounding factors including infant sex, maternal age, birth weight, body length, daily vancomycin dosage, serum albumin (ALB), estimated glomerular filtration rate (eGFR) and serum creatinine (SCr), GAM analysis confirmed a significant nonlinear correlation between GA and initial TCV (Fig. 2).

Two-piecewise linear regression was further applied to quantify the segmented trend. The log-likelihood ratio test revealed *P* = 0.007, which demonstrated that the piecewise regression model possessed markedly superior goodness-of-fit compared with the simple linear model. The inflection point was determined at 34.6 weeks of gestation (approximately 242 days) (Table 5). For neonates with GA less than 34.6 weeks, GA was positively correlated with initial TCV (β = 0.08, 95%CI: 0.01–0.14, *P* = 0.021). By contrast, a marginally significant negative trend was observed when GA exceeded 34.6 weeks (β = −0.10, 95%CI: −0.20 to 0.00, *P* = 0.059).

**Table 5:** Two-Piecewise Linear Regression Model to Evaluate Relationship Between Gestational Age and Initial TCV

	Initial TCV (β, 95% CI, *P*)
Model I	
A straight line effect	0.03 (-0.03, 0.09) 0.284
Model II	
Inflection point (K)	242
< K	0.08 (0.01, 0.14) 0.0213
> K	-0.10 (-0.20, 0.00) 0.0589
P for log-likelihood ratio test	0.007

We adjusted sex, age, weight, length, vancomycin daily dose, ALB, eGFR and SCr

*Abbreviations: TCV* trough concentrations of vancomycin, *ALB* albumin, *SCr* serum creatinine, *eGFR* estimated glomerular filtration rate, *CI* Confidence interval

### Target attainment by gestational age

Among the 143 neonates, 20 (14.0%) had subtherapeutic concentrations (< 5 µg/mL), 103 (72.0%) were within the 5–15 µg/mL target range, and 20 (14.0%) had supratherapeutic concentrations (> 15 µg/mL; Appendix Table 1). The distribution of out-of-range exposure varied across GA categories.

## Discussion

This study elucidated a significant non-linear relationship between gestational age and the initial vancomycin trough concentration, identifying an inflection point at 34.6 weeks. We focused on characterizing the continuous relationship between gestational age and trough concentration values, rather than assessing target attainment rates, due to the ongoing debate surrounding neonatal-specific therapeutic thresholds and the lack of available minimum inhibitory concentration (MIC) data. A two - piecewise linear regression model analysis revealed 34.6 weeks of gestation as the inflection point for trough concentration. To the left of the inflection point (GA < 34.6 weeks), GA exhibited a positive correlation with initial TCV (β = 0.08, 95% CI: 0.01 to 0.14, *P* = 0.021). Conversely, to the right of the inflection point (GA > 34.6 weeks), a negative trend was observed (β = -0.10, 95% CI: -0.20 to 0.00, *P* = 0.059). In patients with a gestational age of less than 34.6 weeks, a lower gestational age was correlated with reduced initial trough concentrations, thereby increasing the risk of subtherapeutic exposure. Conversely, for gestational ages exceeding 34.6 weeks, a closer proximity to term was associated with a decreased likelihood of achieving target trough concentrations. The clinical implications of these pharmacokinetic findings on safety outcomes were not assessed in this retrospective study and warrant further prospective investigation. Additionally, postnatal age (PNA) may affect the interpretation of our gestational age-target concentration variability (GA-TCV) findings [11]. In our cohort, the distribution of PNA was narrow (median 1.0 day, interquartile range 1.0–5.5), which limited the ability to distinguish between the effects of gestational age and postnatal age. Anderson et al. [12] demonstrated that postmenstrual age (PMA) serves as the most effective predictor of clearance, surpassing postnatal age (PNA) in accuracy. Collectively, size, PMA, and renal function account for 82% of the variability in clearance. Within our early-treatment cohort, the influence of PNA may be subsumed by PMA, which is the sum of gestational age (GA) and PNA. Consequently, the inflection point observed at 34.6 weeks may represent a combination of gestational age-dependent developmental processes and postnatal age-dependent maturation.

These findings align with established developmental pharmacokinetic alterations related to gestational age, as documented in the literature. In this study, we examine three pharmacokinetic parameters that may account for the observed pattern, drawing upon well-established pharmacological principles.

### The influence of GA on vancomycin volume of distribution (Vd)

Notably, neonates in our cohort initiated vancomycin therapy at an extremely early postnatal age (median PNA = 1 day, IQR 1.0–5.5 days), which differs markedly from Western studies that mostly collected trough concentrations beyond the first week of life [12]. This ultra-early treatment window is a distinctive feature of domestic clinical practice for suspected early-onset sepsis [13]. Rapid dynamic physiological maturation within the first postnatal week may partially explain the unique nonlinear GA-TCV association observed in our data. Fluctuations in extracellular fluid volume during the initial days of life exert unique effects on vancomycin Vd that cannot be extrapolated to infants receiving vancomycin after postnatal day 7 [9, 10].

Preterm neonates within the first week of life have markedly elevated total body water (85%) and extracellular fluid proportion (60%), values far higher than those seen in late preterm and term infants after postnatal day 7 [14].Vancomycin is a water-soluble antibiotic mainly distributed in extracellular fluid; during the first postnatal week, extracellular fluid volume contracts rapidly, gradually reducing Vd as PNA increases [9]. This rapid contraction of extracellular fluid volume unique to the first week of neonatal life differentiates our PK observations from cohorts with TDM performed beyond postnatal day 7. For neonates younger than 34.6 weeks, expanded extracellular fluid volume elevates Vd and reduces measured trough concentrations. After 34.6 weeks, gradual body fluid contraction offsets this effect, which partially contributes to the negative correlation between GA and TCV above the inflection point.

In this study, the inflection point at 34.6 weeks likely reflects the balance between GA-dependent extracellular fluid expansion and the rapid postnatal contraction occurring during the first week of life. For preterm infants with GA < 34.6 weeks, the expanded extracellular fluid volume in the immediate neonatal period elevates Vd and thereby lowers initial trough concentrations. As GA exceeds 34.6 weeks, the degree of ECF expansion is relatively less pronounced, and postnatal fluid shifts occur more rapidly, which partially contributes to the plateau and subsequent negative correlation between GA and TCV.

Although plasma albumin increases with GA, it was not significantly associated with initial TCV in our cohort (Table 3) and was excluded from the final non-linear models.

### The influence of GA on vancomycin clearance (CL)

Clearance is the principal factor influencing the residence time of a drug within the body. Vancomycin is predominantly eliminated through glomerular filtration, with its clearance being directly associated with glomerular filtration rate (GFR). While the development of neonatal GFR is distinctly dependent on gestational age [15], it also undergoes rapid maturation during the first postnatal week [16].In our cohort, where vancomycin was initiated at a median PNA of 1 day, GFR may still be influenced by perinatal hemodynamic transitions—including the postnatal surge in renal blood flow and cardiac output—that are largely complete by the end of the first week.

Neonates with lower gestational age have immature kidneys, fewer glomeruli, and lower baseline GFR. In this study, the median eGFR in the GA < 34.6 weeks group (31.89 mL/min/1.73 m²) was lower than that in the GA > 34.6 weeks group (37.70 mL/min/1.73 m²). However, within this ultra-early treatment window, the rapid postnatal increase in GFR may already begin to offset GA-dependent immaturity, particularly as neonates approach the 34.6-week inflection point.

The piecewise linear regression analysis demonstrated a declining trend in trough concentrations after 34.6 weeks, consistent with rapidly improving renal clearance during the first postnatal week. For gestational ages exceeding 34.6 weeks, an increase in gestational age is associated with a gradual enhancement in GFR and an accelerated excretion of vancomycin, which is attributed to the progressive maturation of neonatal renal function. Notably, the median daily vancomycin dose was higher (38.46 mg/kg) in those with a gestational age over 34.6 weeks compared to those with a lower gestational age. However, the vancomycin trough concentration did not increase correspondingly, further corroborating the efficient drug clearance facilitated by mature renal systems during this early postnatal period. This early clearance maturation may be less apparent in studies where TDM is performed after the first week, when renal function has largely stabilized.

### Relationship to AUC-guided monitoring

Current guidelines advocate for an AUC₂₄/MIC ratio of ≥ 400 as the primary efficacy target for vancomycin therapy in the treatment of MRSA [1, 17]. In neonatal patients, trough concentrations exhibit a strong correlation with AUC₂₄, rendering trough monitoring a viable surrogate for AUC-guided dosing [9, 10, 18]. Frymoyer et al. [18] evaluated this relationship in 233 neonates and demonstrated that trough concentrations of 7–11 mg/L were highly predictive of AUC/MIC ≥ 400 in more than 90% of patients, although the AUC/MIC varied up to 3-fold for a given trough concentration. Stockmann et al. [19] subsequently externally validated this model in 243 neonates, confirming that a trough of 11 mg/L predicted AUC ≈ 400 in 93% of patients, while noting that trough alone did not precisely predict AUC and reinforcing the need for Bayesian approaches. Chen et al. [20] in a cohort of 213 Chinese neonates, reported that trough concentrations of 5–10 mg/L achieved AUC/MIC ≥ 400 in 95% of infants when the MIC was ≤ 0.5 mg/L, but target attainment fell to only 15% when the MIC increased to 1 mg/L. These findings highlight that the clinical significance of any trough concentration is intrinsically linked to the pathogen’s MIC [21]. For instance, a trough level of 7 µg/mL may be therapeutic for CoNS with an MIC of 0.5 mg/L, yet subtherapeutic for S. aureus with an MIC of 2 mg/L. In the absence of comprehensive MIC data for all enrolled patients, rigid clinical categorization could be misleading [21].

Our identification of a gestational age-dependent inflection point in TCV at approximately 34.6 weeks underscores the necessity for gestational age-specific trough targets when implementing AUC-guided monitoring in neonates [18, 20]. Pham et al. [21] further emphasized that an AUC/MIC target of 400–600 appears to be the most appropriate initial target for vancomycin in neonates, but cautioned that limited clinical outcomes data exist to support the higher end of this range in neonate populations. Additionally, Smits et al. [22] demonstrated that neonatal vancomycin protein binding differs markedly from adults (median fraction unbound 0.9 vs. 0.6), suggesting that a lower total AUC/MIC target may yield equivalent unbound drug exposure in neonates. Consequently, our primary analysis characterized the GA–TCV relationship as a continuous phenomenon. Clinically, target trough concentrations should be individualized within the 5–15 µg/mL range according to infection site, severity, likely pathogens, and MIC values [18, 21, 23].

### Clinical implications

The alignment of our continuous and categorical analyses—particularly the 34.6 weeks inflection point and the gestational age-dependent gradient in subtherapeutic rates—yields distinct clinical management strategies. For GA < 34.6 weeks, low TCV reflects the combined effects of an increased volume of distribution and immature renal clearance, corroborated by the highest subtherapeutic exposure rate (21.2%) in the most preterm group (≤ 29 weeks), which decreased toward the inflection point (13.6% at 29.1–37 weeks) and was lowest beyond it (9.8%). For infants with GA ≤ 29 weeks receiving vancomycin every 24 h—who exhibited the highest subtherapeutic exposure rate (21.2%)---we suggest trough sampling before the third dose (approximately 48 h of age) rather than before the fourth dose (approximately 72 h), coupled with preparedness for dose escalation (increasing the single dose by 20–30% or shortening the interval from 24 to 12 h) if the initial TCV is < 5 µg/mL. For infants on q12h or q8h regimens, in whom trough sampling before the fourth dose already occurs within the first 36–48 h of life, we emphasize ensuring that TDM is not delayed beyond this window, as rapid physiologic maturation during the first postnatal week may render early concentrations non-representative of steady-state exposure. Conversely, for GA ≥ 34.6 weeks, renal clearance accelerates with maturation; despite higher median daily doses, TCV did not increase proportionally. The negative trend beyond the inflection point (β = −0.10, 95% CI: −0.20 to 0.00, *P* = 0.059) suggests an elevated risk of subtherapeutic exposure as neonates near term. Notably, the peak supratherapeutic rate (18.6%) occurred in the 204–259 day group spanning the inflection point, indicating that neonates in this transitional maturity window are particularly vulnerable to overdosing under current weight-based protocols. Across all GA categories, target trough concentrations should be individualized within the 5–15 µg/mL range according to infection site, severity, likely pathogens, and MIC values. These recommendations are hypothesis-generating and require validation in prospective, multicenter studies [24, 25].

## Conclusions

This single-center retrospective study of 143 neonates, who initiated vancomycin at a median postnatal age of 1 day, found a significant non-linear association between gestational age and initial vancomycin trough concentration during the first week of life, identifying an inflection point at approximately 34.6 weeks. To the left of this inflection point, lower gestational age was associated with reduced trough concentrations; to the right, a negative trend of borderline significance was observed. These findings elucidate gestational age-specific patterns in vancomycin exposure during the immediate neonatal period and suggest that existing gestational age-based dosing protocols may not adequately capture the rapid pharmacokinetic transitions occurring in this early window. Pending prospective validation, these results provide a hypothesis-generating foundation for gestational age-stratified therapeutic drug monitoring protocols tailored to the first postnatal week. This study is limited by its single-center retrospective design, small sample size, and narrow postnatal age distribution. Future multicenter prospective studies with larger sample sizes and broader postnatal age ranges are needed to validate these conclusions and to refine individualized vancomycin dosing strategies for neonates.

## Data Availability

The raw data generated and analyzed during this retrospective study are not publicly available to protect neonatal patient privacy and comply with institutional ethical requirements (Ethics Approval No. 2023010). Relevant data can be acquired from the corresponding author upon reasonable request.

## Appendix

**Table 6:** Description of the study population (trough concentrations at different gestational ages

GESTATIONAL_AGE categorical（weeks）	<=29	>29, <=37	>37	P-value	P-value*
N	33	59	51		
VANCO_TROUGH_CONCENTRATIONS	7.50 (2.00-16.30)	10.20 (2.00-46.50)	8.70 (2.90-26.60)	0.101	0.120
VANCO_TROUGH_CONCENTRATIONS categorical				0.406	-
<=5	7 (21.21%)	8 (13.56%)	5 (9.80%)		
>5, <=15	23 (69.70%)	40 (67.80%)	40 (78.43%)		
>15	3 (9.09%)	11 (18.64%)	6 (11.76%)		

Table Results: (N) Mean(SD) Median (Min-Max) / N(%)

## Abbreviations

ALB: Serum albumin
AUC₂₄/MIC: 24-hour area under concentration-time curve/minimum inhibitory concentration ratio
CI: Confidence interval
CoNS: Coagulase-negative staphylococci
DOL: Day of life
eGFR: Estimated glomerular filtration rate
GA: Gestational age
GAM: Generalized additive model
Hb: Hemoglobin
IDSA: Infectious Diseases Society of America
LFT: Liver function test
MAR: Medication administration record
MRSA: Methicillin-resistant Staphylococcus aureus
NEU: Neutrophil count
PMA: Postmenstrual age
PNA: Postnatal age
PPK: Population pharmacokinetics
SCr: Serum creatinine
TCV: Initial vancomycin trough concentration
TDM: Therapeutic drug monitoring
Vd: Volume of distribution
WBC: White blood cell count
